# Quarterly Report

**Published:** 1830-01-01

**Authors:** 


### EDINBURGH SURGICAL HOSPITAL.

QUARTERLY REPORT. By JAS. SYME, Esq.*

We need not say how long and how earnestly we have entreated the medical attendants of provincial hospitals to publish reports of the valuable cases that constantly fall to their charge. Scarcely a Number of this Journal has seen the light for these last four or five years without an exhortation to the gentlemen in question to rouse them from their lethargy, and convince their brethren that they are not altogether like the sleeper Nourjahad. We regret to say that the appeal is not yet answered, as it should be answered from John o' Groat's to the Lizard's Point, with universal activity and zeal. Too many of our country surgeons and physicians still spread the oblivious veil of silence on their practice, and crawl from the cradle to the grave with the proud satisfaction of having done *nothing* for that science, which perhaps did all for them. Is there no mode of stinging into sensibility the callous feelings of these blameably unambitious men, no argument ad hominem that will prick their *ursine* sloth, no spirit-stirring impulse that will quicken into life the dormant generous emulation implanted by kind Nature in the bosom of the dullest? Alas we know of none, except the gradual but steady operation of public opinion, conveyed through the medium of the PRESS. We are happy to be able to exempt not a few provincial surgeons from what may seem the sweeping censure we have uttered. The medical officers of the infirmary at Glasgow, Dr. Ballingall of Edinburgh, and the many respectable and able contributors to the Midland Journal and Provincial Gazette are honourable exceptions, to whom are due, and to whom we have seized every fit opportunity of awarding the unbought praises of independent men. The last who has commenced this goodly race is Mr. Syme, and that under circumstances of rather peculiar character.

It seems, for reasons which he does not state and which perhaps it would be impertinent in others to enquire, that in the early part of this year Mr. S. was induced to hazard the establishment of a surgical hospital in Edinburgh. Minto House was leased, pupils enlisted, patients applied, and better than all *contributors* stepped forward, the end of all which necessary preliminaries was—a hospital of twenty-four beds, "with all appurtenances and means to boot," and a quarterly report in due time from Mr. Syme of all the hair-breadth 'scapes and perils of its inmates. From this report we shall select what is most interesting and begin with :—

I. EXCISION OF THE ELBOW-JOINT.

"This operation was performed twice, viz. on Janet Burns, aged 25, from Carnwath, and on John Wells, aged 9. The mode of procedure was the same as that detailed in the account lately published in this Journal of three cases where the operation was performed.

* Edinb. Journ of Med. Science, No. CI. Oct.

"There was nothing in the previous history of these cases worthy of mention. They both laboured under well-marked caries of the elbow-joint, and would both, a short time ago, have been condemned to amputation without any ceremony. Janet Burns was harrassed by a slight degree of chronic bronchitis, which delayed her recovery, and rendered the complete and permanent establishment of her health somewhat doubtful. She left the Hospital considerably better in this respect than when she entered it, and with the prospect of retaining a useful arm.

"The boy was a most favourable subject for the operation. His disease resulted from external injury, a fall on the elbow, his constitution was good, and he possessed a most excellent disposition, which induced him to perform accurately whatever he was desired in regard to the position and exercise of the limb. Five weeks have now elapsed since the operation, and he is beginning to regain command of the joint, which is nearly as moveable as ever. I expect a most complete recovery in this case, which will be the more remarkable, as a very large portion of the ulna was removed. After sawing off the extremity of the humerus, and cutting away with the pliers the olecranon and head of the radius, I thought from the sound appearance of the different surfaces that enough had been done, and dressed the wound. But it fortunately happened that when the excised portions were afterwards more carefully examined, one of the gentlemen present, Dr. Vallange, observed, that the cut surface of the olecranon presented a small carious cavity, a portion of which must consequently have been allowed to remain. I immediately undid the dressings, and by replacing the olecranon discovered the carious part, which was a sort of cylindrical excavation no wider than a common quill, but running deeply into the bone. Having ascertained its extent by introducing a probe, I insulated the ulna as far as was necessary, and cut it across through the shaft, so as to detach the whole spongy portion of the bone, which was then removed, though not without some difficulty, owing to the connexion of the brachiæus internus.

"In this case the only muscle left undisturbed was the biceps; and the difficulty of moving the joint ought to have been if possible still greater than some people allege it to be, when merely the triceps is detached from its insertion. It has surprised me considerably to find that my pupils felt it difficult to conceive how the efficiency of the muscles could be restored in these circumstances, since there are so many parallel cases of every day's occurrence, for instance, the use of a stump, which is so soon regained, owing to the muscles fixing themselves round the bone.

"The difficulty of conceiving this very easy matter was, however, so great, that I requested Mr. Y———, whose case is detailed in my former paper on this subject, to allow the gentlemen attending my clinical lectures to satisfy themselves by ocular and manual examination of the very perfect command which he was able to exercise over his arm. This gentleman is preparing to finish his education as a clergyman, and finds himself able not only to write sermons, but to execute all the ordinary motions of the arm."

### II. EMPLOYMENT OF THE ACTUAL CAUTERY.

Every body knows, or ought to know, that there is a periodical mania in surgery and medicine for the re-introduction of ancient practice and obsolete opinions. The rabies of the anti-contagionists comes regularly round after a term of years, and so it is with many other things, especially with the employment of the actual cautery. Heaven knows this measure had a pretty fair trial, and was not abandoned until after its merits had been tested by a long and dreadful experience. Gentlemen, however, now-a-days would wish to persuade us that a red-hot iron is a pleasant thing enough, and by no means attended with those disagreeable sensations with which its application is invested in the eyes of vulgar prejudice. Mr. Syme has for some time past made much use of the cautery as a counterirritant in that scape-goat for fifty diseases "the morbus coxarius," and "the *similar* affection of the shoulder-joint, the *omalgia* of Rust." It is much to be lamented that gentlemen will still make use of unmeaning terms like the above, terms which embrace so many and such various maladies of the parts in question, that the published results of any mode of treatment employed for them is not worth one farthing!

III. Excision of the Mamma.

"Mary Messer, æt. 38, from Torwoodlee, had been afflicted for nearly three years with all the symptoms so well described by Sir A. Cooper under the title of irritable tubercle of the breast. About two years ago she consulted me on account of these complaints, when I recommended the use of means proper for restoring the uterine secretions, which had long been very irregular, and for three months previous to that time altogether suppressed. She complied with these directions, and in the course of a week had a return of the interrupted discharges. Her complaints were then much alleviated, and continued to be so for several months, when, though the uterine actions continued regular, the symptoms of her complaint became considerably aggravated, and at length the almost incessant, occasionally most unsufferable, pain of her breast shooting into the arm, shoulder, and side, tormented her so grievously both night and day, that she resolved on having the disease removed by the knife. With this view she was sent to the Surgical Hospital by my friend Dr. Anderson of Selkirk.

"Conceiving it right to comply with the patient's urgent desire to have the breast excised, since all other means of relief had failed, and success had attended extirpation of the testicle when similarly affected, I performed the operation on the 13th May. The wound healed by the first intention, and she left the Hospital on the 23d, quite free from her former sufferings, and in a state of mind very different from the extreme dejection and anxiety which characterised it previous to the operation. According to the latest accounts from Dr. Anderson she continues perfectly well. The breast on dissection exhibited the appearances described by Sir A. Cooper, being merely more dense and uniform in structure than usual.

Janet Anderson, æt. 40, entered the Hospital on the 8th May, on account of a scirrhous mamma, which had recently suppurated to a small extent on the surface; it was removed a few days afterwards, the wound healed by the first intention, and she would have been dismissed as quickly as the last mentioned patient, but an abscess formed in the axilla, which excited our worst suspicions, and induced us to detain her for some time longer. Fortunately this abscess did not turn out to be malignant, but healed most satisfactorily, and the patient was dismissed quite well on the 18th June. She returned a few days ago to show that she continued free from complaint, and offer thanks for the care which had been bestowed upon her, previously to departure for the north.

"Margaret Mathieson, aged 23, was recommended to the hospital on the 10th of June by Dr. Johnston of Kirkaldy, on account of a very large and exceedingly hard tumour in the axilla. It filled the axillary cavity so completely as to prevent the arm from being approximated to the side, and was occasionally the seat of severe lancinating pain. It had existed more than half a year, and was continuing to increase progressively. Notwithstanding the youth of the patient, the symptoms just mentioned would probably have induced me to remove the tumour, had other circumstances been favourable to this proceeding, but it was rendered quite impracticable by the firm connexions of the tumour, and even if this objection could have been overcome, the existence of many hard tumours of a smaller size in the neck and throat would have rendered an operation quite unjustifiable.

"It occurred to me, that, as the uterine discharges were suspended, advantage might result from the internal administration of cantharides, especially as this medicine has a very remarkable effect in promoting the action of the absorbent vessels in general. In no long time after commencing the course prescribed to her, she noticed a remarkable diminution not only of the pain, but also of the swelling, and regularly improved until the 10th of July, when, being comparatively speaking well, she returned home in great joy at her recovery."

We would remark that of the many cases of irritable or hysterical breast which we have witnessed not one *demanded* amputation. It is a remedy which we should resort to with the utmost possible reluctance, for even if not succeeded by dangerous symptoms, the irritability or pain may re-appear in the cicatrix, the other breast, or perhaps in a distant part, to the bitter disappointment of the patient, and regret, perhaps disgrace of the surgeon. We hold an operation in such cases to be bad practice, except in very particular circumstances.

IV. HYDROCELE.

"There were only two cases of hydrocele, but both rather interesting. The first was that of William Mackintosh, æt. 28, a north country cattle drover, who entered the Hospital on the 17th May, labouring under the following complication of diseases :—Sores on the penis, bubo, ague caught on passing through some of the fenny districts in England, and a hydrocele of nine years standing. Having subdued his other disorders, I punctured the hydrocele, and evacuated a large quantity of chocolate-coloured fluid, holding in suspension many of those small shining scales which my friend Dr. Christison has found to be Cholesterine. As there was much enlargement of the testicle, and great thickening of the sac, we did not think it right to inject, and proposed to the patient to perform either the old operation of excising the sac, that is to say, a portion of it, or the more simple process of castration. He preferred the latter, but before submitting to it, found it necessary to return to the north to execute some business of importance. It is this sort of hydrocele which has been named hæmatocele, and probably with some reason. In the case just related it was observed, that when the dark brown fluid was allowed to stand quietly in the glass, a quantity of pure blood collected in the bottom, and in another case formerly under my care, the hemorrhagic nature of the disease was still more manifest. I punctured a large hydrocele, and drew off a quantity of the same sort of fluid as above described; but finding that by far the greater part of the swelling still remained, and that the patient, who for several years has been frequently prevented by fits of pain from following his avocation for weeks together, was now suffering more than ever, I proposed removal of the testicle, and performed the operation with perfect success. On examining the tumour, I was not a little surprised to find the testicle quite sound, and that what had led me to think it enlarged was a great mass of dense fibrinous matter, which adhered no less firmly to the tunica vaginalis than the coagulum of an old aneurism does to its inner surface.

"The other case of hydrocele treated in the Hospital was that of Alexander Wood, aged 24,—a well marked case of hydrocele of the cord. I drew off the contents which were perfectly pale and limpid, but did not inject, since it seems that dropsy in this situation is not so apt to return after evacuation as when it is seated in the tunica vaginalis.

"The patient accordingly had a very slight return of the swelling, which soon subsided, and he has been dismissed cured."

Our want of space compels us to stop, but we recommend Mr. Syme to prosecute the system of reporting the interesting cases that occur to him. By doing so candidly and conscientiously he will confer a boon on the profession, he will earn a laurel, or possibly a more substantial reward, for himself. Were we allowed, in conclusion, to hint a fault in the present report, it were that of *curing* the patients too rapidly. When Mr. Syme is somewhat older as a hospital surgeon he will learn the fallacies of hospital practice, and find that "dismissed cured," is oftentimes a rather deceptive record. We part from Mr. S. with much respect.

